# Phenotypic Detection of Carbapenem Resistance in Klebsiella pneumoniae, Acinetobacter baumannii, Pseudomonas aeruginosa, and Enterobacter Species at a Tertiary Care Hospital

**DOI:** 10.7759/cureus.109319

**Published:** 2026-05-20

**Authors:** Sujata P Korochikar, Priyanka M Mane, Satish R Patil, Yogendra P Shelke, Suvarna N Patil, Premkumar Korochikar

**Affiliations:** 1 Department of Microbiology, Krishna Institute of Medical Sciences, Krishna Vishwa Vidyapeeth (Deemed to be University), Karad, IND; 2 Department of Microbiology, B.K.L. Walawalkar Rural Medical College, Chiplun, IND; 3 Department of Medicine, B.K.L. Walawalkar Rural Medical College, Chiplun, IND; 4 Department of Advanced Diploma in Medical Laboratory Technology, Shri Vithalrao Joshi Charities Trust’s, College of Advanced Studies, Chiplun, IND

**Keywords:** acinetobacter baumannii, carbapenem resistance, cbde, ecim, enterobacter species, klebsiella pneumoniae, mcim, pseudomonas aeruginosa

## Abstract

Background

Carbapenem resistance in Gram-negative *Enterococcus faecium*, *Staphylococcus aureus*, *Klebsiella pneumoniae*, *Acinetobacter baumannii*, *Pseudomonas aeruginosa*, and *Enterobacter* species (ESKAPE) pathogens has emerged as a significant concern in hospital settings, particularly in developing regions, due to its association with multidrug resistance and limited therapeutic options. The present study was designed to assess the prevalence, specimen-wise distribution, and phenotypic detection of carbapenemase production among these pathogens in a tertiary care setting using Clinical and Laboratory Standards Institute (CLSI) M100, 33rd edition, 2023, recommended methods, including the modified carbapenem inactivation method (mCIM) and the EDTA-modified carbapenem inactivation method (eCIM).

Methodology

This is a prospective observational study conducted at a tertiary care hospital in southwest Maharashtra from January to December 2024. A total of 312 non-duplicate Gram-negative ESKAPE pathogens isolated from various clinical samples were included. Identification and antimicrobial susceptibility testing were performed according to the CLSI M100, 33rd edition, 2023 edition. Carbapenem-resistant isolates were further analyzed for the detection of carbapenemase production by the phenotypic method, mCIM, and eCIM.

Results

Among the 312 Gram-negative ESKAPE isolates studied, 94 (30.13%) were identified as carbapenem-resistant. *K. pneumoniae* accounted for the largest proportion of carbapenem-resistant isolates, 41/94 (43.62%), whereas the highest species-specific carbapenem resistance rate was observed in *A. baumannii,* with 23/57 (40.35%) isolates carbapenem-resistant. Urine was the most frequent source of specimens (54.24%). Carbapenemase production was detected in 62/94 (65.96%) carbapenem-resistant isolates by mCIM, and among these, 39/62 (62.90%) were identified as metallo-β-lactamase (MBL) producers by eCIM. High resistance rates were observed against cephalosporins, fluoroquinolones, and aminoglycosides. In the study setting, colistin resistance was 8/94 (8.51%), with higher resistance in *P. aeruginosa*, 4/23 (17.39%).

Conclusions

A considerable burden of carbapenem-resistant Gram-negative ESKAPE pathogens was observed, with the majority producing carbapenemases and a significant proportion producing MBLs. The presence of multidrug resistance and emerging resistance to last-line agents is concerning. Strengthening routine carbapenemase detection, infection control practices, and antimicrobial stewardship programs is essential.

## Introduction

*Enterococcus faecium*, *Staphylococcus aureus*, *Klebsiella pneumoniae*, *Acinetobacter baumannii*, *Pseudomonas aeruginosa*, and *Enterobacter* species (ESKAPE) are clinically important organisms recognized for their ability to survive antimicrobial pressure and contribute to healthcare-associated infections. This study focuses on four Gram-negative ESKAPE bacteria: *K. pneumoniae*, *A. baumannii*, *P. aeruginosa*, and *Enterobacter* species. These bacteria are particularly associated with multidrug resistance and severe hospital-acquired infections [1]. Carbapenems are extensively employed as highly effective agents for treating infections caused by multidrug-resistant Gram-negative bacteria; however, increasing carbapenem resistance has emerged as a significant clinical challenge. According to the Clinical and Laboratory Standards Institute (CLSI) guidelines M100, 33rd edition, 2023, resistance to carbapenems is defined as decreased susceptibility to at least one carbapenem, such as imipenem, meropenem, or ertapenem. The main mechanism driving this resistance is the production of carbapenemase enzymes, which are categorized into Ambler classes A, B, and D [2]. *Enterobacter* species and *K. pneumoniae* are associated with multiple carbapenemases, including *K. pneumoniae* carbapenemase, metallo-β-lactamases (MBLs), and oxacillinase-48 (OXA)-like enzymes. In contrast, *A.baumannii* is predominantly linked to class D-type OXA-type carbapenemase. *P. aeruginosa* frequently produces MBLs in addition to intrinsic resistance mechanisms [2,3]. Reliable detection of carbapenemase production is crucial for guiding appropriate antimicrobial therapy and ensuring effective infection control. In accordance with the recommendations of the CLSI M100, 33rd edition, 2023, phenotypic techniques, including the modified carbapenem inactivation method (mCIM), are used for detecting carbapenemase activity, whereas the ethylene diamine tetraacetic acid (EDTA)-modified carbapenem inactivation method (eCIM) is used to identify MBLs. However, these methods do not differentiate between serine carbapenemase (Ambler classes A and D) [4]. Increasing reports from Indian tertiary care centers indicate a rising prevalence of carbapenemase-producing Gram-negative pathogens, posing significant therapeutic and infection-control challenges [5].

In this context, the present study was undertaken to evaluate carbapenem resistance among Gram-negative ESKAPE pathogens. The specific objectives were to determine the prevalence of carbapenem-resistant isolates, assess carbapenemase production using phenotypic methods, and analyze their distribution across different clinical specimens.

## Materials and methods

Ethical approval and study design

Ethical clearance for the study was obtained from the Institutional Ethics Committees of Krishna Institute of Medical Sciences, Krishna Vishwa Vidyapeeth (Deemed to be University), Karad (approval number: KIMSDU/IEC/04/2023), and B.K.L.Walawalkar Rural Medical College (approval number: BKLWRMC/IEC/43/2023). This was a prospective, observational, laboratory-based study conducted in the Department of Microbiology of a tertiary care hospital over one year, from January 2024 to December 2024.

Sample size

A consecutive inclusion approach was followed, in which all eligible, non-duplicate, Gram-negative ESKAPE isolates obtained during the study period were analyzed. A total of 312 isolates were included in the study to assess antimicrobial resistance.

Study population and sample collection

The study included non-duplicate, clinically significant isolates of Gram-negative ESKAPE bacteria, namely, *K. pneumoniae*,* A. baumannii*,* P. aeruginosa*,* and Enterobacter *species, obtained from specimens of patients admitted to inpatient departments (IPDs). Repeated isolation of the same organism with an identical antibiogram from the same patient within 14 days was excluded. Isolates considered to be colonizers or contaminants were not included. Both adult and pediatric patients were included in the study.

Identification of bacteria and antimicrobial susceptibility testing

Clinical specimens, such as blood, urine, respiratory samples, and wound swabs, were processed according to standard microbiological procedures. Blood samples were initially incubated in the BACT/ALERT automated blood culture system (bioMérieux, Marcy l’étoile, France) to facilitate pathogen recovery. Positive blood culture specimens and other clinical specimens were inoculated onto blood agar and MacConkey agar (HiMedia Laboratories Pvt. Ltd., Mumbai, India) and incubated aerobically at 37°C for 24 hours. Bacterial Identification was performed using conventional biochemical methods, including Gram staining, oxidase test, catalase test, indole production, citrate utilization, urease test, triple sugar iron agar reactions, and motility testing. Antimicrobial susceptibility testing was performed using the Kirby-Bauer disk diffusion method on Mueller-Hinton agar (HiMedia Laboratories Pvt. Ltd., Mumbai, India) in accordance with CLSI M100, 33rd edition, 2023 recommendations. Antibiotic disks used in this study included amikacin (30 µg), ciprofloxacin (5 µg), ceftazidime (30 µg), cefepime (30 µg), fosfomycin (200 µg), nitrofurantoin (300 µg), meropenem (10 µg), imipenem (10 µg), cotrimoxazole (1.25/23.75 µg), and piperacillin-tazobactam (100/10 µg) (HiMedia Laboratories Pvt. Ltd., Mumbai, India). Nitrofurantoin and fosfomycin were tested only for urinary isolates.

Colistin susceptibility testing

Colistin susceptibility testing was performed using the colistin broth disk elution (CBDE) method in accordance with the CLSI M100, 33rd edition, 2023 guidelines. For each isolate, four tubes containing 10 mL of cation-adjusted Mueller-Hinton broth (HiMedia Laboratories Pvt. Ltd., Mumbai, India) were prepared. The tubes corresponded to colistin concentrations of 0, 1, 2, and 4 µg/mL, achieved by adding 0, 1, 2, and 4 colistin disks (10 µg each) (HiMedia Laboratories Pvt. Ltd., Mumbai, India), respectively. The tubes were kept at room temperature for at least 30 minutes to ensure adequate elution of colistin. A bacterial suspension adjusted to 0.5 McFarland was prepared, and 50 µL was inoculated into each tube. The tubes were incubated for 16-20 hours at 35°C. After incubation, tubes were examined for visible turbidity, and the lowest colistin concentration at which no growth was observed was recorded as the minimum inhibitory concentration (MIC). Results were interpreted as intermediate (≤2 µg/mL) or resistant (≥4 µg/mL) [6]. Quality control was ensured using *P. aeruginosa* American Type Culture Collection (ATCC) 27853 and *Escherichia coli* ATCC) 25922. CBDE was considered the reference method, and a comparison between CBDE results and those obtained from the automated VITEK 2 system was performed [6].

Detection of carbapenemase production

Carbapenemase production was evaluated using mCIM, in accordance with CLSI M100, 33rd edition, 2023 guidelines [6]. The test organism was inoculated into tryptic soy broth (HiMedia Laboratories Pvt. Ltd., Mumbai, India), and a 10 µg meropenem disk was added, followed by incubation at 35°C for four hours. Subsequently, the disk was transferred onto Mueller-Hinton agar (HiMedia Laboratories Pvt. Ltd., Mumbai, India) seeded with a 0.5 McFarland suspension of *E. coli* (ATCC 25922) and incubated overnight. A reduced inhibition zone around the disk indicated carbapenemase production. Results were interpreted as follows: an inhibition zone diameter of 6-15 mm was considered positive; 16-18 mm (with pinpoint colonies) was considered intermediate (interpreted as positive); and ≥19 mm was considered negative [6].

Detection of metallo-β-lactamase

mCIM-positive isolates were further evaluated for MBL production using eCIM, following CLSI M100, 33rd edition, 2023 guidelines [6-8]. EDTA (HiMedia Laboratories Pvt. Ltd., Mumbai, India), a metal-chelating agent, was added to tryptic soy broth containing the test organism along with a meropenem (10 µg) disk (HiMedia Laboratories Pvt. Ltd., Mumbai, India), and the mixture was incubated for four hours at 35°C. The disk was subsequently transferred onto Mueller-Hinton agar (HiMedia Laboratories Pvt. Ltd., Mumbai, India) inoculated with a 0.5 McFarland suspension of *E. coli* (ATCC 25922) and incubated overnight. An increase in zone diameter of ≥5 mm in eCIM compared with mCIM alone was considered indicative of MBL production, whereas an increase of ≤4 mm was interpreted as negative, as per CLSI M100, 33rd edition, 2023 recommendations [6,7].

Data collection and statistical analysis

Relevant demographic and clinical variables, such as age, sex, hospital unit, and specimen type, were documented. The collected data were analyzed using descriptive statistical methods, including the proportion and distribution of carbapenem-resistant and carbapenemase-producing isolates.

## Results

Demographic and specimen distribution of Gram-negative ESKAPE pathogens

During the study period, a total of 312 Gram-negative ESKAPE pathogens were obtained from various clinical specimens. Among these, 94 (30.13%) isolates were identified as carbapenem-resistant. The largest proportion of patients was male, accounting for 65 (69.15%) of cases, while females constituted 29 (30.85%). Most isolates were derived from patients admitted to the IPD, 52 (55.32%), while the remaining 42 (44.68%) were from the intensive care unit (ICU). Table 1 shows that carbapenem-resistant Gram-negative ESKAPE pathogens were more frequently isolated from urine samples, accounting for 32 (54.24%) of all carbapenem-resistant isolates. Among urine specimens, *K. pneumoniae*, 14 (23.73%), was the most frequently identified organism, followed by *P. aeruginosa*, 10 (16.95%), *A. baumannii*, 5 (8.47%), and *Enterobacter* species, 3 (5.08%). These findings indicate that the urinary tract may serve as a significant reservoir for carbapenemase-producing organisms in hospital settings. Pus and wound samples constituted the second most common source, 27 (32.14%), with *A. baumannii*, 11 (13.10%), identified as the predominant isolate, followed by *K. pneumoniae*, 7(8.33%), and *P. aeruginosa*, 6 (7.14%). These observations reinforce the contribution of *A. baumannii* in wound-related and postoperative infections. Blood and body fluid samples accounted for 12 (26.09%) isolates, with *K. pneumoniae*, 6 (13.04%), as the most common organism, followed by *A. baumannii*, 4 (8.70%), and *P. aeruginosa*, 2 (4.35%), while *Enterobacter* species isolates were not obtained from these specimens. Respiratory samples showed the lowest proportion, 23 (18.70%), with *K. pneumoniae*, 14 (11.38%), being the predominant organism, followed by *P. aeruginosa*, 5 (4.07%), and *A. baumannii*, 3 (2.44%).

**Table 1:** Specimen-wise distribution of carbapenem-resistant Gram-negative ESKAPE pathogens. Data are presented as number (percentage). Percentages for each organism are calculated with respect to the total number of isolates within each specimen type. Total percentage represents the proportion of carbapenem-resistant isolates among all specimens processed (n = 312). *Enterobacter species* include *Enterobacter cloacae* complex and related species. Respiratory samples include sputum, endotracheal aspirate, and bronchoalveolar lavage. Blood and other body fluid specimens include blood, cerebrospinal fluid, pleural fluid, and sterile-site specimens. ESKAPE = *Enterococcus faecium*, *Staphylococcus aureus*, *Klebsiella pneumoniae*, *Acinetobacter baumannii*, *Pseudomonas aeruginosa*, and *Enterobacter* species

Specimen type	*Klebsiella pneumoniae,* n (%)	*Enterobacter species,* n (%)	*Pseudomonas aeruginosa,* n (%)	*Acinetobacter baumannii, *n (%)	Total, n (%)
Urine (n = 59)	14 (23.73)	3 (5.08)	10 (16.95)	5 (8.47)	32 (54.24)
Respiratory (n = 123)	14 (11.38)	1 (0.81)	5 (4.07)	3 (2.44)	23 (18.70)
Pus/Wound (n = 84)	7 (8.33)	3 (3.57)	6 (7.14)	11 (13.10)	27 (32.14)
Blood/Body fluids (n = 46)	6 (13.04)	0 (0.00)	2 (4.35)	4 (8.70)	12 (26.09)
Total (n = 312)	41 (13.14)	7 (2.24)	23 (7.37)	23 (7.37)	94 (30.13)

Distribution of carbapenem-resistant ESKAPE isolates

Table 2 presents the prevalence and distribution of carbapenem-resistant Gram-negative ESKAPE isolates among different organisms. *A. baumannii* showed the highest species-specific prevalence of carbapenem resistance, with 23/57 (40.35%) isolates being carbapenem-resistant, followed by *K. pneumoniae*, 41/121 (33.88%), *P. aeruginosa*, 23/94 (24.47%), and *Enterobacter *species, 7/40 (17.50%). However, among the total carbapenem-resistant isolates, *K. pneumoniae* was the most common organism, accounting for 41 (43.62%) cases. *P. aeruginosa* and *A. baumannii* each accounted for 23 (24.47%), while *Enterobacter *speciescontributed the least at 7 (7.45%).

**Table 2:** Prevalence and distribution of carbapenem-resistant Gram-negative ESKAPE isolates. Data are presented as the number of carbapenem-resistant isolates over total isolates tested (n/N), with percentage in parentheses. Carbapenem resistance is defined as reduced susceptibility to at least one carbapenem agent (imipenem, meropenem, or ertapenem) as per Clinical and Laboratory Standards Institute guidelines. Proportion among total carbapenem-resistant isolates (%) represents the proportion of each organism among the total carbapenem-resistant isolates (n = 94). *Enterobacter species* include *Enterobacter cloacae* complex and related species. ESKAPE = *Enterococcus faecium*, *Staphylococcus aureus*, *Klebsiella pneumoniae*, *Acinetobacter baumannii*, *Pseudomonas aeruginosa*, and *Enterobacter* species

Organism	Carbapenem-resistant, n/N (%)	Proportion among total carbapenem-resistant isolates (%)
*Klebsiella pneumoniae*	41/121 (33.88)	43.62
*Enterobacter species*	7/40 (17.50)	7.45
*Pseudomonas aeruginosa*	23/94 (24.47)	24.47
*Acinetobacter baumannii*	23/57 (40.35)	24.47
Total	94/312 (30.13)	100

Phenotypic detection of carbapenemase production

Table 3 shows the phenotypic distribution of carbapenemase-producing and MBL-producing carbapenem-resistant Gram-negative isolates. During the study period, a total of 312 Gram-negative ESKAPE isolates were obtained. Among these, 94 (30.13%) were carbapenem-resistant by disk diffusion and were included in further analysis. Of these 94 isolates, 62 (65.96%) were positive for production of carbapenemase by mCIM. The highest proportion of mCIM positivity was observed in *K. pneumoniae*, 36/41 (87.80%), followed by *P. aeruginosa*, 20/23 (86.96%), and *Enterobacter* species, 6/7 (85.71%). Among the isolates positive for mCIM, 39 (62.90%) were MBL positive by eCIM. Production of MBL was detected in 22 (61.11%) of mCIM-positive *K. pneumoniae*, 13 (65.00%) of *P. aeruginosa*, and 4 (66.67%) of *Enterobacter* species. *A. baumannii* were not subjected to mCIM and eCIM testing in accordance with CLSI M100, 33rd edition, 2023 guidelines [6].

**Table 3:** Carbapenemase and MBL detection among carbapenem-resistant isolates. *: eCIM was performed only on mCIM-positive isolates for the detection of MBL production. MBL = metallo-β-lactamase; eCIM = EDTA-carbapenem inactivation method; mCIM = modified carbapenem inactivation method; NA = not applicable

Organism	Total, n	mCIM-positive, n (%)	eCIM-positive (MBL), n (%)*
*Klebsiella pneumoniae*	41	36 (87.80)	22 (61.11)
*Pseudomonas aeruginosa*	23	20 (86.96)	13 (65.00)
*Enterobacter species*	7	6 (85.71)	4 (66.67)
*Acinetobacter baumannii*	23	NA	NA
Total	94	62 (65.96)	39 (62.90)

**Figure 1:**
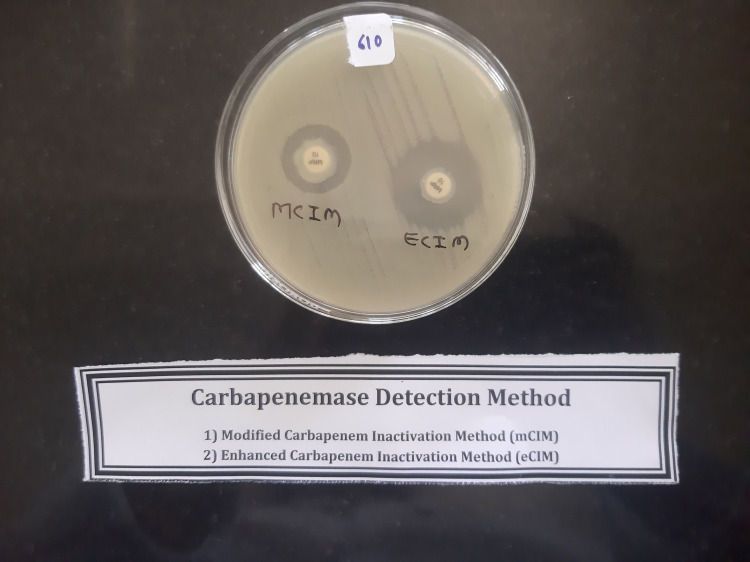
Phenotypic method for carbapenemase detection: mCIM and eCIM. mCIM = modified carbapenem inactivation method; eCIM = EDTA-modified carbapenem inactivation method

Representative images of mCIM and eCIM showing zone diameter differences used for interpretation of carbapenemase and MBL production, as per CLSI guidelines, are shown in Figure 1.

Antimicrobial resistance profile

**Figure 2:**
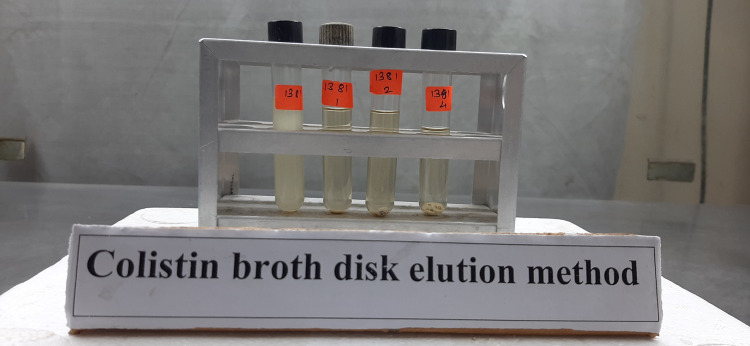
Colistin broth disk elution test.

Table 4 presents 94 carbapenem-resistant Gram-negative ESKAPE pathogens. Resistance to carbapenems was nearly complete, with imipenem resistance observed in 23 (100%) *A. baumannii*, 23 (100%) *P. aeruginosa*, 7 (100%) *Enterobacter* species, and 40 (97.56%) *K. pneumoniae* isolates. Meropenem resistance was observed in all isolates 94 (100%). *A. baumannii* showed complete resistance, 23 (100%), to cefepime, ceftazidime, ciprofloxacin, meropenem, imipenem, and piperacillin-tazobactam, along with high resistance to amikacin, 21 (91.3%), and cotrimoxazole, 17 (73.91%). *P. aeruginosa* also exhibited complete resistance to carbapenems, 23 (100%), with resistance rates of 16 (69.57%) to ceftazidime and cefepime, 17 (73.91%) to ciprofloxacin, 19 (82.61%) to piperacillin-tazobactam, and 15 (65.22%) to amikacin. *K. pneumoniae* demonstrated very high resistance, including 41 (100%) resistant to meropenem and piperacillin-tazobactam, 40 (97.56%) to ceftazidime and cefepime, and 39 (95.12%) to amikacin and ciprofloxacin, while resistance to cotrimoxazole was 28 (68.29%). Similarly, *Enterobacter* species showed complete resistance of 7 (100%) to cephalosporins, carbapenems, ciprofloxacin, and piperacillin-tazobactam, with cotrimoxazole resistance of 6 (85.71%). Colistin resistance remained low overall, 8/94 (8.51%), although higher colistin resistance was detected in *Pseudomonas* species, 4 (17.39%), and *Enterobacter* species, 1 (14.28%), compared to *A. baumannii*, 1 (4.35%), and *K. pneumoniae*, 2 (4.88%). Among urinary isolates, fosfomycin resistance was 4/14 (28.57%) in *K. pneumoniae* and 2/3 (66.66%) in *Enterobacter* species, while nitrofurantoin resistance was 10/14 (71.43%) and 2/3 (66.66%), respectively. Figure 2 illustrates the CBDE method employed for assessing susceptibility to colistin.

**Table 4:** Antibiotic resistance pattern of carbapenem-resistant ESKAPE pathogens. *: Nitrofurantoin and fosfomycin susceptibility were tested only in urinary isolates (denominator varies). ESKAPE = *Enterococcus faecium*, *Staphylococcus aureus*, *Klebsiella pneumoniae*, *Acinetobacter baumannii*, *Pseudomonas aeruginosa*, and *Enterobacter* species; NA = not applicable

Antibiotic	*Acinetobacter baumannii* (n = 23), n (%)	*Pseudomonas aeruginosa* (n = 23), n (%)	*Klebsiella pneumoniae* (n = 41), n (%)	*Enterobacter species* (n = 7), n (%)
Amikacin	21 (91.30)	15 (65.22)	39 (95.12)	5 (71.43)
Ceftazidime	23 (100)	16 (69.57)	40 (97.56)	7 (100)
Ciprofloxacin	23 (100)	17 (73.91)	39 (95.12)	7 (100)
Colistin	1 (4.35)	4 (17.39)	2 (4.88)	1 (14.28)
Cefepime	23 (100)	16 (69.57)	40 (97.56)	7 (100)
Fosfomycin*	NA	NA	4/14 (28.57)	2/3 (66.66)
Nitrofurantoin*	NA	NA	10/14 (71.43)	2/3 (66.66)
Imipenem	23 (100)	23 (100)	40 (97.56)	7 (100)
Meropenem	23 (100)	23 (100)	41 (100)	7 (100)
Cotrimoxazole	17 (73.91)	NA	28 (68.29)	6 (85.71)
Piperacillin–tazobactam	23 (100)	19 (82.61)	41 (100)	7 (100)

## Discussion

The current study demonstrates that 30.13% (94/312) of Gram‑negative ESKAPE isolates are carbapenem-resistant, indicating a substantial burden of carbapenem‑resistant Gram‑negative pathogens in our tertiary care hospital. This prevalence falls within the 25-40% range reported for carbapenem‑resistant Gram‑negative ESKAPE organisms in recent Indian and global surveillance, highlighting the growing threat of carbapenem‑resistant infections in both ICU and IPD settings [8,9]. The male predominance of 65 (69.15%) aligns with Indian‑based studies [8,9]. The predominance of urinary isolates and wound-related samples suggests an important role for urinary infections in the dissemination of resistant organisms, consistent with Indian and global reports linking carbapenem‑resistant *K. pneumoniae* and *P. aeruginosa* to complicated urinary tract infections [8-11]. Blood and body fluid samples contributed 12 (26.09%) isolates, dominated by *K. pneumoniae*, 6 (13.04%), and *A. baumannii*, 4 (8.70%), while respiratory specimens contributed 23 (18.70%) isolates, with *K. pneumoniae* being the most frequently isolated organism. Overall, in this study setting, urine and wound‑related specimens were the primary sources of carbapenem‑resistant isolates, with *A. baumannii* and *K. pneumoniae* emerging as the most frequent ESKAPE pathogens across specimen types, reinforcing the need for targeted infection control and antimicrobial stewardship in urological, surgical, and critical‑care areas [8,9].

Table 2 indicates differences between species-specific prevalence of carbapenem resistance and the overall distribution of carbapenem-resistant isolates. *A. baumannii* showed the highest species-specific prevalence of carbapenem resistance, with 23/57 (40.35%) isolates being carbapenem-resistant, followed by *K. pneumoniae* at 41/121 (33.88%), *P. aeruginosa* at 23/94 (24.47%), and *Enterobacter* species at 7/40 (17.50%). However, among the total carbapenem-resistant isolates (n = 94), *K. pneumoniae* accounted for the largest proportion, contributing 41 (43.62%) isolates, whereas *A. baumannii* and *P. aeruginosa* each accounted for 23 (24.47%) isolates. Similar findings have been reported in previous studies, in which *K. pneumoniae* accounted for the largest number of resistant isolates, despite higher species-specific resistance rates in *A. baumannii* [10,11]. In the present study, 94/312 (30.13%) of Gram-negative ESKAPE isolates were carbapenem-resistant, indicating a considerable burden in the clinical setting. Among these, 62/94 (65.96%) were confirmed as carbapenemase producers by mCIM. The highest proportion of carbapenemase-producing isolates was observed in *K. pneumoniae*, followed by *P. aeruginosa* and *Enterobacter* species, suggesting their important role in hospital-associated resistance. Phenotypic findings further suggested that among the carbapenemase-producing isolates, 39/62 (62.90%) were identified as MBL producers by eCIM. MBLs are zinc-dependent enzymes that hydrolyze carbapenems and are associated with limited therapeutic options [2]. A subset of carbapenem-resistant isolates tested negative by mCIM, suggesting alternative resistance mechanisms, including efflux pump overexpression, porin loss, or AmpC β-lactamases and extended-spectrum β-lactamases, coupled with reduced membrane permeability [12]. *A. baumannii* was not evaluated for carbapenemase detection by mCIM/eCIM due to known methodological limitations, which should be considered when interpreting these findings.

Antimicrobial susceptibility testing revealed near‑complete carbapenem resistance (≥97-100% to imipenem and meropenem) across all ESKAPE‑group organisms, with high resistance to third‑generation cephalosporins, fluoroquinolones, and β‑lactam/β‑lactamase‑inhibitor combinations, mirroring the extensive multidrug‑resistant profile reported from other Indian ESKAPE surveillance series [9,10]. Overall, colistin resistance in the study setting remained low, with resistance observed in 8/94 (8.51%) isolates. However, higher colistin resistance rates were noted in *P. aeruginosa*, 4 (17.39%), and *Enterobacter* species, 1 (14.28%), raising concern regarding emerging resistance to this last-line agent, a trend also reported in recent Indian studies on colistin resistance [8,10]. In urinary isolates, fosfomycin and nitrofurantoin showed moderate-to-high resistance rates in *K. pneumoniae* and *Enterobacter* species, suggesting reduced efficacy of these traditionally used urinary agents against carbapenem-resistant Gram-negative uropathogens. This aligns with Indian data documenting rising fosfomycin and nitrofurantoin non-susceptibility among carbapenem‑resistant Gram‑negative urinary isolates, suggesting the need for updated empiric and targeted treatment strategies for urinary tract infections in the era of carbapenem resistance [8,10]. Collectively, these findings indicate a considerable burden of carbapenem-resistant Gram-negative ESKAPE pathogens in the hospital setting studied. This emphasizes the urgent need for routine carbapenemase detection (mCIM/eCIM), robust infection‑control measures, and strong antimicrobial stewardship in Indian hospitals, consistent with current Indian and global guidance on carbapenem‑resistant organisms [8,10-12].

Limitations

The present study has certain limitations, including its single-center design, relatively small sample size, absence of molecular characterization of resistance genes, and limited epidemiological analysis. In addition, *A. baumannii* was not evaluated by mCIM/eCIM due to the known methodological limitations of these phenotypic methods in this organism. These factors may limit the broader generalizability and detailed characterization of resistance mechanisms.

## Conclusions

This study highlights a substantial burden of carbapenem-resistant Gram-negative ESKAPE pathogens, with the majority of carbapenem-resistant isolates producing carbapenemases, including a significant proportion of MBL producers. The high level of resistance to carbapenems and other commonly used antibiotics reflects the narrowing of effective treatment options. Although colistin remained active against most isolates, emerging resistance to last-resort agents is concerning. These findings emphasize the need for routine phenotypic carbapenemase detection, strengthened infection-control measures, and effective antimicrobial stewardship programs to limit the spread of antimicrobial resistance.
